# Pathway Engineering, Re-targeting, and Synthetic Scaffolding Improve the Production of Squalene in Plants

**DOI:** 10.1021/acssynbio.2c00051

**Published:** 2022-05-13

**Authors:** Jacob D. Bibik, Sarathi M. Weraduwage, Aparajita Banerjee, Ka’shawn Robertson, Roberto Espinoza-Corral, Thomas D. Sharkey, Peter K. Lundquist, Björn R. Hamberger

**Affiliations:** † Cell and Molecular Biology Program, Michigan State University, East Lansing, Michigan 48824, United States; ‡ DOE Great Lakes Bioenergy Research Center, Michigan State University, East Lansing, Michigan 48824, United States; § Department of Biochemistry and Molecular Biology, Michigan State University, East Lansing, Michigan 48824, United States; ∥ DOE Plant Research Laboratory, Michigan State University, East Lansing, Michigan 48824, United States; ⊥ The Plant Resilience Institute, Michigan State University, East Lansing, Michigan 48824, United States

**Keywords:** squalene, lipid droplet scaffolding, plastid targeting, plastid membrane scaffolding

## Abstract

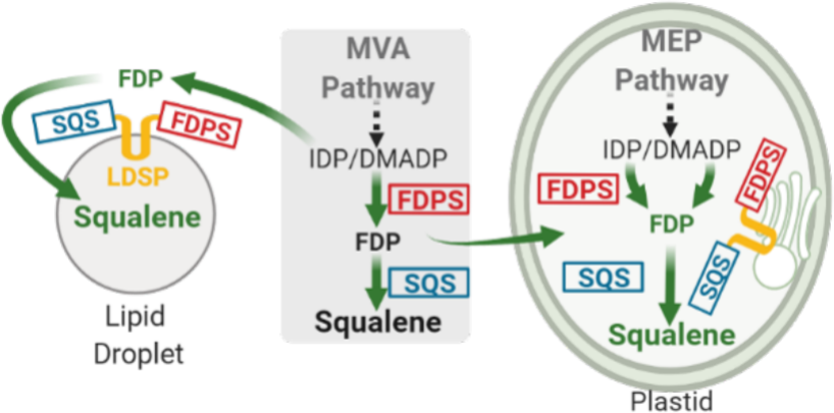

Plants are increasingly becoming an option for sustainable bioproduction of chemicals and complex molecules like terpenoids. The triterpene squalene has a variety of biotechnological uses and is the precursor to a diverse array of triterpenoids, but we currently lack a sustainable strategy to produce large quantities for industrial applications. Here, we further establish engineered plants as a platform for production of squalene through pathway re-targeting and membrane scaffolding. The squalene biosynthetic pathway, which natively resides in the cytosol and endoplasmic reticulum, was re-targeted to plastids, where screening of diverse variants of enzymes at key steps improved squalene yields. The highest yielding enzymes were used to create biosynthetic scaffolds on co-engineered, cytosolic lipid droplets, resulting in squalene yields up to 0.58 mg/gFW or 318% higher than a cytosolic pathway without scaffolding during transient expression. These scaffolds were also re-targeted to plastids where they associated with membranes throughout, including the formation of plastoglobules or plastidial lipid droplets. Plastid scaffolding ameliorated the negative effects of squalene biosynthesis and showed up to 345% higher rates of photosynthesis than without scaffolding. This study establishes a platform for engineering the production of squalene in plants, providing the opportunity to expand future work into production of higher-value triterpenoids.

## Introduction

Engineered plants present an opportunity for sustainable production of high-value chemicals important for many industries. One class of chemicals with growing interest is terpenoids, the most diverse class of natural products with an array of biotechnological applications. The C_30_ triterpene squalene is a long-chain hydrocarbon and the precursor to sterols and triterpenoids.^1^ Since first being described in shark liver oil in 1916,^2^ it has been developed for a number of commercial uses that include in cosmetic oils, as a vaccine adjuvant, and has potential as an energy dense biofuel.^3,4^ Squalene is also an important intermediate in the production of higher-value derivatives, such as the triterpenoid ambrein and its derivative (−)-ambrox, which are used in the fragrance industry.^5−7^ For commercial applications, squalene has historically been obtained from the shark liver and more recently vegetable oils,^8^ but engineered crops may be able to produce it with higher specificity and yields. Establishing more sustainable plant production strategies for squalene and the derived triterpenoids may enable an economically viable platform for supply to a range of industries. It has also been suggested that incorporating engineered biosynthetic pathways into bioenergy crops may improve the financial feasibility of both terpenoid production and conversion of plant biomass to biofuels.^9^ Using plants to produce squalene and valuable derivatives requires innovation to increase yields while reducing potentially negative effects of engineered pathways on the host.^10−12^

**Figure 1:**
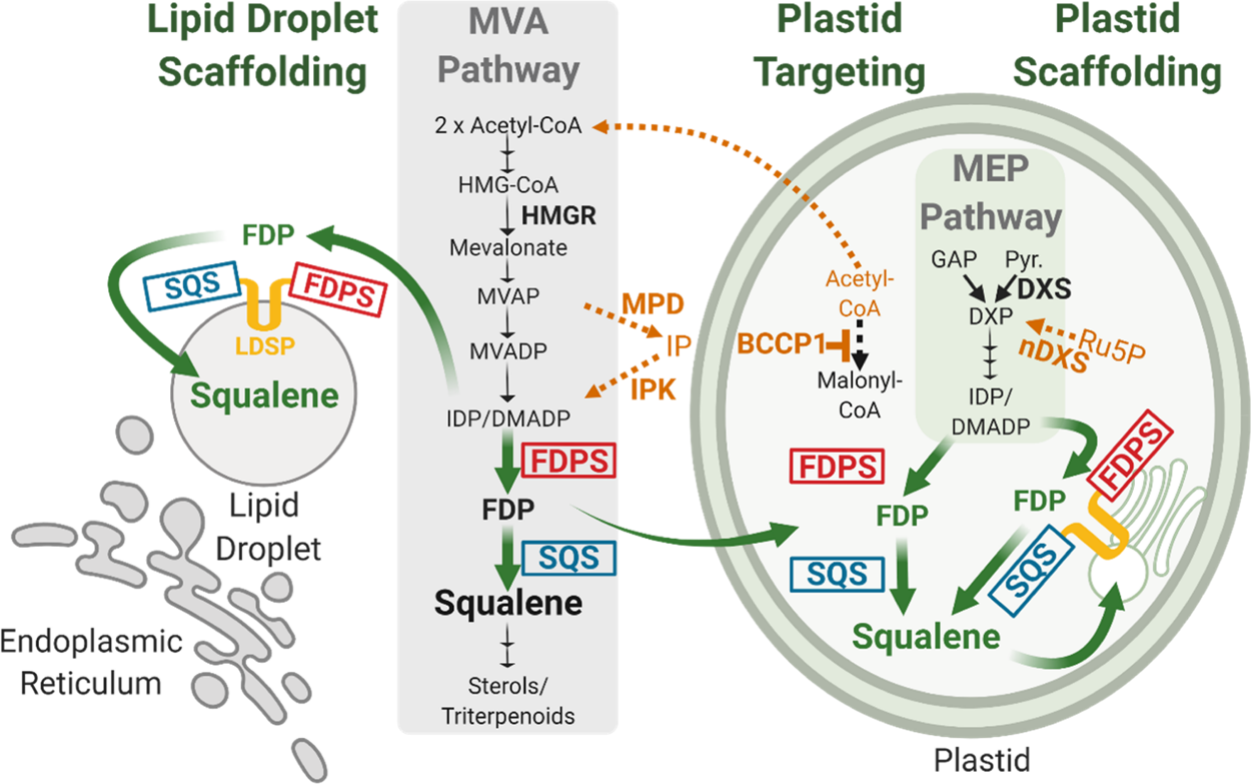
Overview of the engineering strategies developed or tested to improve squalene production in plants. Green arrows indicate the engineered squalene pathways developed in this study, scaffolding pathways on cytosolic lipid droplets (left), or re-targeting to plastids (right) with and without scaffolding. Orange pathways indicate alternative contribution strategies tested, where genes were co-expressed with genes for various squalene pathways.

The terpene backbones from which terpenoids are derived are assembled in five carbon segments through condensation of the building blocks isopentenyl diphosphate (IDP) and dimethylallyl diphosphate (DMADP).^13^ In plants, these building blocks are synthesized either in the cytosol through the mevalonate (MVA) pathway, starting with condensation of 2 acetyl-CoA molecules, or in plastids through the methylerythritol 4-phosphate (MEP) pathway, starting with pyruvate and glyceraldehyde 3-phosphate (GAP) condensation (Figure 1). Also localized to the cytosol is farnesyl diphosphate synthase (FDPS), which catalyzes the head-to-tail condensation of one DMADP and two IDP molecules to form the C15 farnesyl diphosphate (FDP). Two of these FDP molecules are then condensed head-to-head by the endoplasmic-reticulum-bound squalene synthase (SQS) to form squalene. Multiple approaches have been taken to increase terpenoid yields in plants through engineering or re-targeting these pathways.

Both the MVA and MEP pathways are regulated through multiple mechanisms, many of which have become targets for engineering.^13−18^ Common strategies to overcome regulatory limitations include overexpression and engineering of key enzymes in both pathways. Overexpression of the genes for 1-deoxy-d-xylulose-5-phosphate synthase (DXS), the first step in the MEP pathway, and 3-hydroxy-3-methylglutaryl-CoA reductase (HMGR), the committed step to the MVA pathway (Figure 1), has been shown to generate abundant supply of terpenoid precursors IDP and DMADP while increasing terpenoid yields.^19−22^ Both DXS and HMGR have been targets for engineering, where variants have been created and shown to have reduced negative regulation in plant systems.^23,24^ Other studies have indicated alternative contributions to the precursor pathways or that IDP and DMADP pools can also improve terpenoid production.^22,25,26^ Overexpression of the *Arabidopsis thaliana* biotin carboxyl carrier protein 1 (*At*BCCP1) gene was shown to improve acetyl-CoA availability and utilization by the MVA pathway to increase terpenoid yields.^22^ Addition of a phosphomevalonate decarboxylase from *Roseiflexus castenholzii* (*Rc*MPD) and an *Arabidopsis* isopentenyl phosphate kinase (*At*IPK), a non-canonical route to IDP using MVA pathway intermediates, was also found to improve terpenoid production in plants.^25,26^ These studies provide potential biological parts for optimization or combinatorial approaches to further develop plant systems for terpenoid production.

Re-targeting and compartmentalization of terpenoid pathways have enabled the storage of products to increase yields^27−29^ and reduce the negative effects of product accumulation on plants.^30,31^ Previous work has shown that re-directing terpene biosynthesis from the cytosol to plastids in plants using the organelle as a storage compartment can increase yields.^27,28,30^ In other works, lipid droplets have been adapted as synthetic storage compartments for terpenes and terpenoids, increasing yields further.^29,31^ Co-production of terpenes and lipid droplets was shown to not only increase terpene yields but also enable a platform for bioproduction of both terpenes and other lipids of interest, such as triacylglycerols of which the lipid droplets are composed. These co-production strategies for terpenoids and lipid droplets may further improve the economic feasibility of bioproduction hosts, which has become a focus with the green alga *Haematococcus pluvialis* producing the tetraterpenoid astaxanthin and triacylglycerols.^32,33^

In this work, a multi-pronged approach was taken to advance plants as a production platform for squalene and triterpenoid derivatives. A series of enzyme screenings were performed to optimize plastidial-targeted squalene biosynthesis using both native and engineered enzyme variants. The lipid droplet surface protein from *Nannochloropsis oceanica* (*No*LDSP)^34^ was used to anchor the optimized squalene pathway to the surface of cytosolic lipid droplets in different variations, synthesizing squalene at the surface of lipid droplets and increasing yields. Next, the lipid droplet scaffolding strategy was re-targeted from the cytosol to plastids, where scaffolding occurred on membranes throughout chloroplasts and ameliorated the negative effects of squalene accumulation on photosynthesis. Finally, combinations of *AtIPK*, *RcMPD*, and *AtBCCP1* were co-expressed with cytosolic and lipid droplet squalene pathways in attempts to boost yields further.

## Results and Discussion

### Screening to Improve the Entry Step in the MEP Pathway

**Figure 2:**
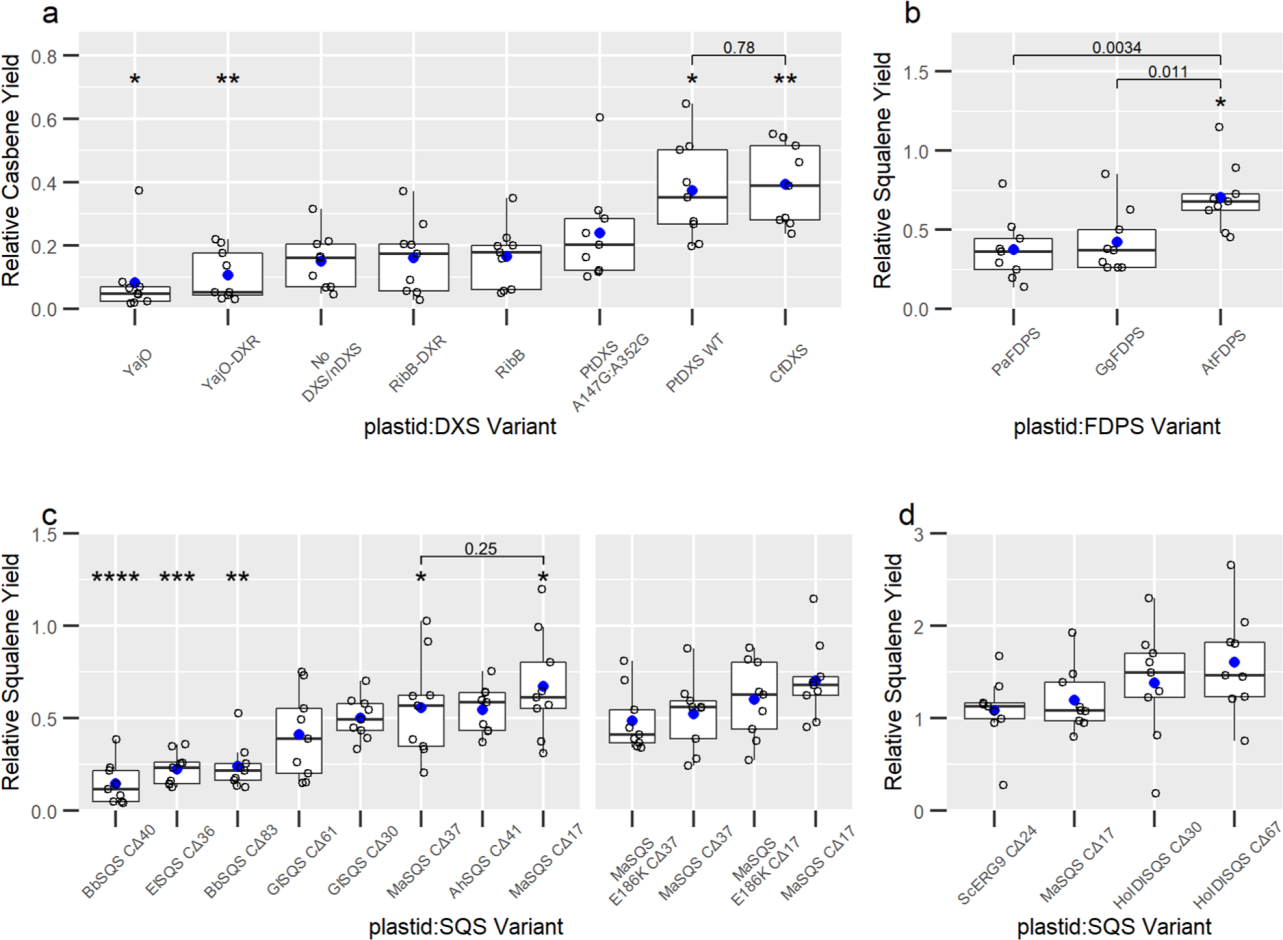
Plastid-targeted pathway optimization for squalene production. Screening of key steps in plastid localization for DXS and nDXS (a), FDPS orthologues (b), truncated SQS orthologues (c, left), *Ma*SQS mutants (c, right), and additional SQS variants (d). Each panel presents data collected from separate transient expression experiments, except panel (c, right), which was from the same experiment as panel (b), while panel (c, left) was a separate experiment. Open circles are individual data points, blue circles are mean values, and the horizontal line within the box represents the median value. The box shows the range from the lower 25th percentile to the upper 75th percentile. The upper and lower whiskers extend to the largest and smallest data point no further than 1.5× the inter-quartile range, with points lying outside the whiskers considered outliers. Asterisks indicate a significant difference of the mean relative squalene yield for each variant compared to the mean of all variants in that experiment based on the *t*-test. “*”: *p* ≤ 0.05; “**”: *p* ≤ 0.01; “***”: *p* ≤ 0.001; “****”: *p* ≤ 0.0001. Individual statistical comparisons between means are shown by brackets and the indicated *p*-value.

The entry step in the MEP pathway synthesizes 1-deoxy-d-xylulose-5-phosphate (DXP) from pyruvate and GAP, catalyzed by DXS (Figure 1). With this step being a major limiting step in the MEP pathway,^14^ controlling flux through the pathway,^19^ as well as being feedback inhibited by the end products IDP and DMADP,^18^ it has become a target for increasing terpenoid production. Previous work has created novel, bacterial DXS-like enzymes (nDXSs) to synthesize DXP from an alternative substrate^35^ or mutate DXS enzymes from poplar to de-regulate and reduce feedback inhibition.^24^ The nDXSs were shown to complement *dxs* knockout lines of *Escherichia coli* grown on xylose as the sole carbon source, synthesizing DXP from ribulose 5-phosphate (Ru5P) (Figure 1). Furthermore, it was shown that fusing the nDXSs to the next enzyme in the MEP pathway, DXS reductoisomerase (DXR), further increased flux through the pathway in *E. coli*.^35^ In studying DXS feedback inhibition, another study found that a double mutant from a *Populus trichocarpa* DXS (*Pt*DXS A147G:A352G) had reduced feedback inhibition by IDP and DMADP in vitro*.*^24^ In addition to reduced feedback inhibition, *Pt*DXS A147G:A352G showed reduced activity in vitro, but this was never tested in plants to determine whether the reduced feedback inhibition can overcome the reduced activity when overexpressed. The two most successful nDXS enzymes from *E. coli*, RibB G108S and YajO, the *P. trichocarpa* double mutant *Pt*DXS A147G:A352G, the wild-type *Pt*DXS, and a DXS from *Coleus forskohlii* (*Cf*DXS) (Table S1) were included in this study. In attempts to overcome limitations at this entry step, introduce novel contributions to the MEP pathway, and increase terpene production in plants, the native and mutant DXSs, and bacterial nDXSs, alone and fused with DXR, were screened for the highest terpene yields (Figure 2a).

While the plant DXS enzymes contain a native plastid transit peptide, a transit peptide sequence from the *A. thaliana* Rubisco small subunit^36^ was added to the N-termini of the bacterial nDXSs, targeting these proteins to plastids. Using *Agrobacterium*-mediated, transient expression in *Nicotiana benthamiana*, each candidate gene was co-expressed along with a geranylgeranyl diphosphate synthase gene from *C. forskohlii* (*CfGGDPS*) and a casbene synthase gene from *Daphne genkwa* (*DgCasS*) (Table S1) to synthesize the diterpene casbene as a proxy for flux toward a terpene product. While *Cf*DXS and *Pt*DXS demonstrated the highest casbene yields, *Pt*DXS A147G:A352G showed reduced yields and the nDXSs did not show any increase over control lines of CasS and GGDPS only (Figure 2a).

*Cf*DXS, the highest-yielding DXS, increased casbene yields by 163% over control lines and showed 80.9% higher yields than the mean of all DXS and nDXS variations (Figure 2a). The wild-type *Pt*DXS showed 72% higher casbene yields than the mean of all variations and showed 57% higher yields than *Pt*DXS A147G:A352G. While Banerjee et al.^24^ demonstrated that *Pt*DXS A147G:A352G had reduced feedback inhibition, they also concluded that the enzyme had reduced activity in vitro. The experiments here suggest the reduction in feedback inhibition does not overcome the reduced enzyme activity to increase terpene yields in plants. The nDXSs did not increase casbene yields, although the substrate, Ru5P, is typically present in chloroplasts as an intermediate of the Calvin–Benson cycle.^37^ While the nDXSs may provide an alternative route capable of complementing a DXS knockout in *E. coli*, nDXS gene overexpression did not result in more terpene accumulation than overexpression of the other wild-type DXS variants in *N. benthamiana*. The wild-type *Cf*DXS resulted in the highest relative casbene yields and was used in subsequent engineering and screening of FDPS and SQS candidates to improve plastidial squalene yields.

### SQS and FDPS Screening to Improve Squalene Yields

While overexpression of key genes involved in the MVA or MEP pathways improves general terpenoid production, optimizing the downstream reactions toward specific products further increases yields.^38,39^ Here, screening of diverse orthologs and engineered variants of FDPS and SQS enabled selection of an optimal combination for squalene production. Six of the orthologous SQS genes were codon-optimized for expression in *N. benthamiana* from the following organisms: *Amaranthus hybridus* (*AhSQS*), *Botryococcus braunii* (*BbSQS*), *Euphorbia lathyris* (*ElSQS*), *Ganoderma lucidum* (*GlSQS*), *Mortierella alpina* (*MaSQS*), and *Saccharomyces cerevisiae* (*ScERG9*), which are all species that can accumulate large amounts of squalene-related compounds. Additionally, a mutant of *MaSQS E186K* was created, which was previously shown to improve the catalytic efficiency 3.4-fold in *in vitro* studies.^40^ Finally, two truncated SQS variants from the diatom *Haslea ostrearia* (*HoIDISQS*), which is a native fusion gene encoding an isopentenyl diphosphate isomerase (IDI) fused to the N-terminus of the SQS, were included. Both protein domains of *Ho*IDISQS were shown to be functional,^41^ and previous studies have shown that co-expression of the gene encoding IDI, which catalyzes interconversion of IDP and DMADP, increases terpene yields.^41,42^ Screenings of SQS and FDPS variants were performed with plastid targeting to compartmentalize squalene accumulation and avoid influence on native, cytosolic squalene biosynthesis.

To target SQS candidates to plastids, first, the predicted C-terminal signal peptide which anchors the protein to the endoplasmic reticulum was removed to solubilize the protein. Each candidate was truncated by two different lengths based on amino acid sequence alignment (truncation length indicated by Δ). The larger truncation was 10 amino acids following the end of conserved homology between sequences, and the shorter eliminated about half the number of amino acids. The same *A. thaliana* transit peptide was then added to the N-termini of truncated variants, targeting solubilized SQS candidates to plastids. SQS candidates were co-expressed with plastid-targeted *FDPS* from *A. thaliana* (*AtFDPS*) and *CfDXS*; then, squalene yields were measured, which are reported as a ratio of squalene to the internal standard, hexacosane (Figure 2c). Expression of only plastid-targeted *AtFDPS* and *CfDXS* resulted in squalene levels similar to the background, indicating little influence on cytosolic squalene production (Figure S1). Candidate FDPS genes were compared from three species: *A. thaliana*, *Picea abies*, and *Gallus gallus* (*N. benthamiana* codon-optimized; accessions given in Table S1). Each FDPS gene was co-expressed with *CfDXS* and *MaSQS CΔ17*, and squalene was measured (Figure 2b).

Comparing FDPS variants, *At*FDPS had statistically significant higher yields than both *Pa*FDPS and *Gg*FDPS (*p* < 0.01 and *p* < 0.05, respectively), which was 41% higher than the mean of all FDPS variants (Figure 2b). Compared to the mean squalene yield of all variants, *Bb*SQS CΔ40, *Bb*SQS CΔ83, and *El*SQS CΔ36 had statistically significant lower yields (*p* < 0.001, *p* < 0.05, and *p* < 0.05, respectively), while *Ah*SQS CΔ41 and *Ma*SQS CΔ17 had significant higher yields (*p* < 0.05) (Figure 2c). The second *Ah*SQS truncation, *Ah*SQS CΔ20, was compared to *Ma*SQS CΔ17, and both resulted in similar yields (Figure S1a). The *Ho*IDISQS-truncated variants were screened alongside *Ma*SQS CΔ17 and the commonly used yeast SQS, *Sc*ERG9, which showed a slight, but not significant, increase in squalene yields (Figure 2d). Although these are not directly comparable without creating IDI fusions with other variants, these fusions may be worth further investigation in future engineering. While the *Ma*SQS CΔ17 E186K variant previously demonstrated an increased catalytic efficiency (*k*_cat_/*K*_m_) in vitro*,*^40^ it did not increase squalene yields with either truncation variant in this system (Figure 2c). *Ma*SQS CΔ17 showed squalene yields 63% higher than the mean yield of other variants (Figure 2c) and was chosen as the SQS to use for development of lipid droplet scaffolding, along with *At*FDPS.

### Lipid Droplet Scaffolding Optimization

Previous work demonstrated that terpene synthases can be targeted to the surface of lipid droplets by fusing the enzymes to *No*LDSP, where terpenoids are stored within the lipid droplets.^29^ Unlike other commonly used lipid droplet proteins like oleosins and seipins which can also be used for scaffolding,^43,44^*No*LDSP has no known plant orthologs.^34^ Additionally, *No*LDSP does not rescue oleosin functions of triacylglycerol turnover, possibly due to a lack of species–specific protein recruitment, which may be favorable in the context of lipid droplet overproduction.^34^ Here, *At*FDPS and *Ma*SQS CΔ17 were used to re-localize squalene biosynthesis to the surface of cytosolic lipid droplets through fusions to *No*LDSP. SQS and FDPS gene variants were co-expressed with the gene coding for a truncated form of HMGR to reduce feedback inhibition from *E. lathyris* (*El*HMGR^159-582^), which was previously shown to drive flux through the MVA pathway and increase cytosolic terpenoid yields.^29^ To increase lipid droplet formation, the gene for a C-terminal, truncated WRINKLED1 transcription factor from *A. thaliana* (*At*WRI1^1-397^) was co-expressed, which has been shown to activate fatty acid biosynthetic pathways and oil production.^45,46^ Co-expression of the genes for *At*WRI1 and *No*LDSP, with or without fusions to terpene synthases, induces accumulation of lipid droplets,^29,34^ which is utilized here to overproduce and functionalize lipid droplets as synthetic organelles. It was previously demonstrated that terpenes are effectively sequestered within lipid droplets when the biosynthetic pathways are anchored to the surface *No*LDSP fusions.^29^

**Figure 3:**
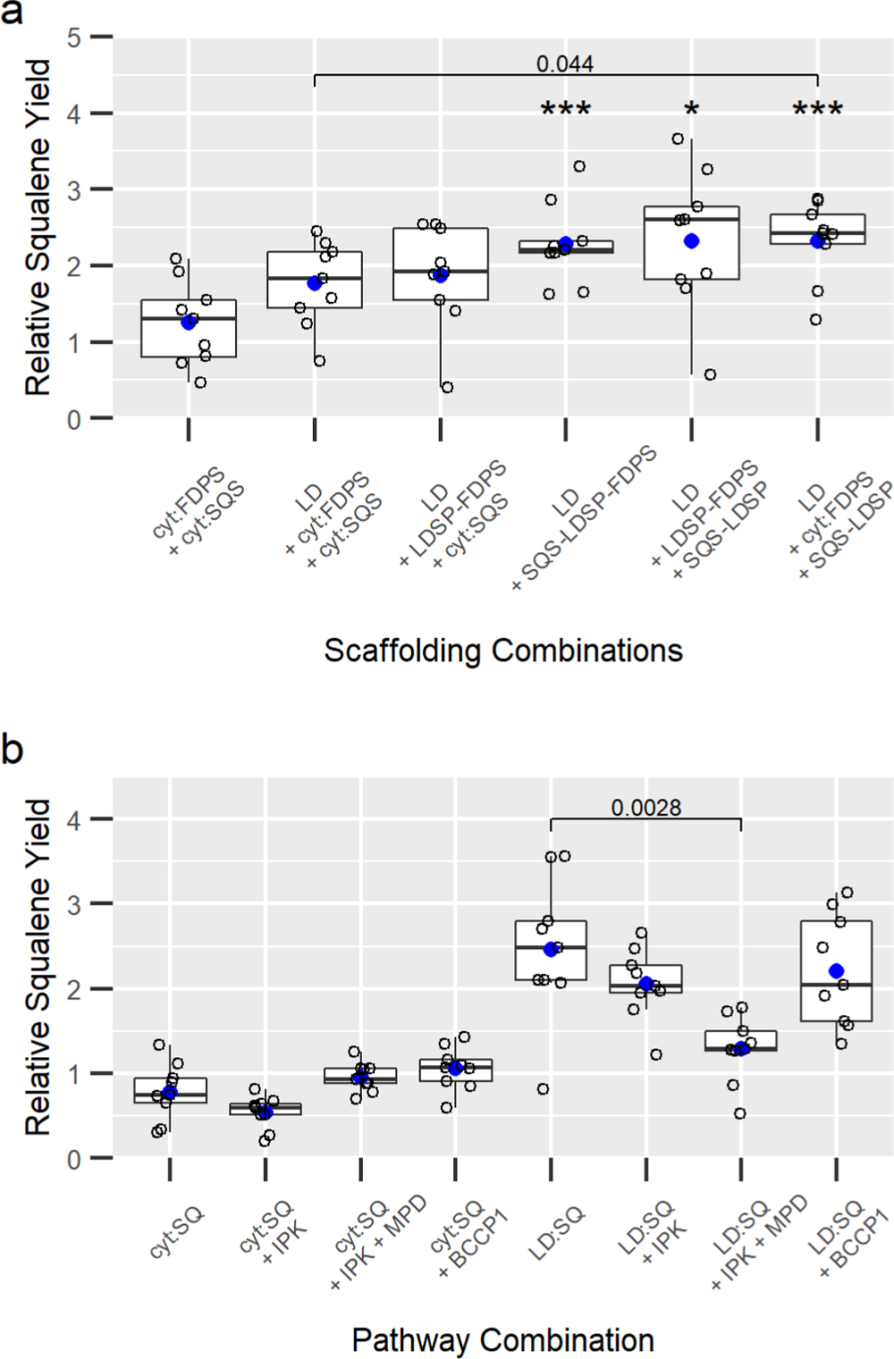
Screening of lipid droplet scaffolding combinations (a) and compatibility of alternative pathway contributions to engineered squalene pathways in the cytosol (b). In (a), all samples are co-expressed with *ElHMGR*^159-582^ and samples include “LD” if they co-express *AtWRI1*^1-397^ and either *NoLDSP* alone or with the indicated *NoLDSP* fusion. In (b), “SQ” indicates co-expression of *ElHMGR*^159-582^ + cyt:*AtFDPS* + cyt:*MaSQS* and “LD:SQ” indicates *ElHMGR*^159-582^ + *AtWRI1*^1-397^ + SQS-LDSP-FDPS. Open circles are individual data points, blue circles are mean values, and the horizontal line within the box represents the median value. The box shows the range from the lower 25th percentile to the upper 75th percentile. The upper and lower whiskers extend to the largest and smallest data points no further than 1.× the inter-quartile range, with points lying outside the whiskers considered outliers. Statistical significance compared to the mean of the cytosolic squalene pathway without lipid droplet scaffolding (*El*HMGR^159-582^ + cyt:FDPS + cyt:SQS) based on the *t*-test is indicated by an asterisk: “*”: *p* ≤ 0.05; “***”: p ≤ 0.001. Individual comparisons between variables are indicated by brackets with the corresponding *p*-value.

Several fusion variants were created to target *At*FDPS and *Ma*SQS CΔ17, together or separately, to lipid droplets (Figures 3a and S1c). Replacing the SQS endoplasmic reticulum retention signal, the N-terminus of *No*LDSP was fused to the C-terminus of the truncated SQS (SQS-LDSP). Since FDPS enzymes are natively soluble, LDSP was initially fused to either the N- or C-terminus of *At*FDPS (LDSP-FDPS or FDPS-LDSP, respectively). The FDPS-LDSP fusion demonstrated reduced yields (Figure S1c), possibly because of interference of the C-terminal fusion located near the active site, according to the crystal structure of the human FDPS.^47^ Therefore, all other combinations used variations of *No*LDSP fused to the C-terminus of *Ma*SQS CΔ17 and the N-terminus of *At*FDPS.

Each fusion protein and soluble cytosolic versions of SQS and FDPS (cyt:SQS and cyt:FDPS) were used to test co-production of squalene and lipid droplets. The co-production of lipid droplets (*At*WRI1 + *No*LDSP) with the soluble, cytosolic pathway *El*HMGR^159-582^ + cyt:SQS + cyt:FDPS increased mean squalene yields by 41%, with a similar increase of 49% when only FDPS was anchored to lipid droplets as LDSP-FDPS (Figure 3a). A significant increase in squalene yields of more than 80% was observed in lipid droplet scaffolding combinations involving SQS fusions to LDSP, which included cyt:SQS-LDSP + cyt:FDPS, cyt:SQS-LDSP + cyt:LDSP-FDPS, and cyt:SQS-LDSP-FDPS.

These data suggest that it is key for the final step in the pathway, SQS, to be anchored to the surface of lipid droplets to increase yields. This may enable direct lipid droplet interactions for the squalene as it is synthesized. Co-localization of both FDPS and SQS at the surface of the droplets resulting in significant yield increases demonstrates a method to create synthetic organelles, which may be an effective strategy to direct biosynthesis further toward higher-value, squalene-derived triterpenoids, or other classes of products. Manipulation of the lipid droplet architecture may provide an additional route to further modify scaffolding and production.

### Targeting LDSP Scaffolds to Plastids

Further sub-compartmentalization of pathways within plastids may provide another strategy to re-direct accumulation of products like squalene.^31^ The SQS-LDSP-FDPS fusion protein was targeted to plastids through the addition of a transit peptide to determine whether the pathway would remain functional if scaffolded in plastids. A plasmid was created where plast:*CfDXS*, plast:*AtFDPS*, and plast:*MaSQS CΔ17* are each separated by an LP4/2A linker^48,49^ (pDFS). The LP4/2A is a hybrid linker which combines post-translational cleavage of LP4 with the co-translational “cleavage” of 2A, allowing expression of a single transcript while producing separate protein products. When compared to pDFS, co-expression of plast:*CfDXS* with the fusion of plast:*SQS-LDSP-FDPS* resulted in similar squalene yields (Figure S1b). To determine if the plast:SQS-LDSP-FDPS fusion was successfully targeted to plastids, vectors were constructed to use EYFP and an EYFP-*No*LDSP fusion, both with and without the plastid transit peptide. A series of experiments were then performed to determine which chloroplast associated membranes the plast:EYFP-*No*LDSP fusion proteins were localizing to.

**Figure 4:**
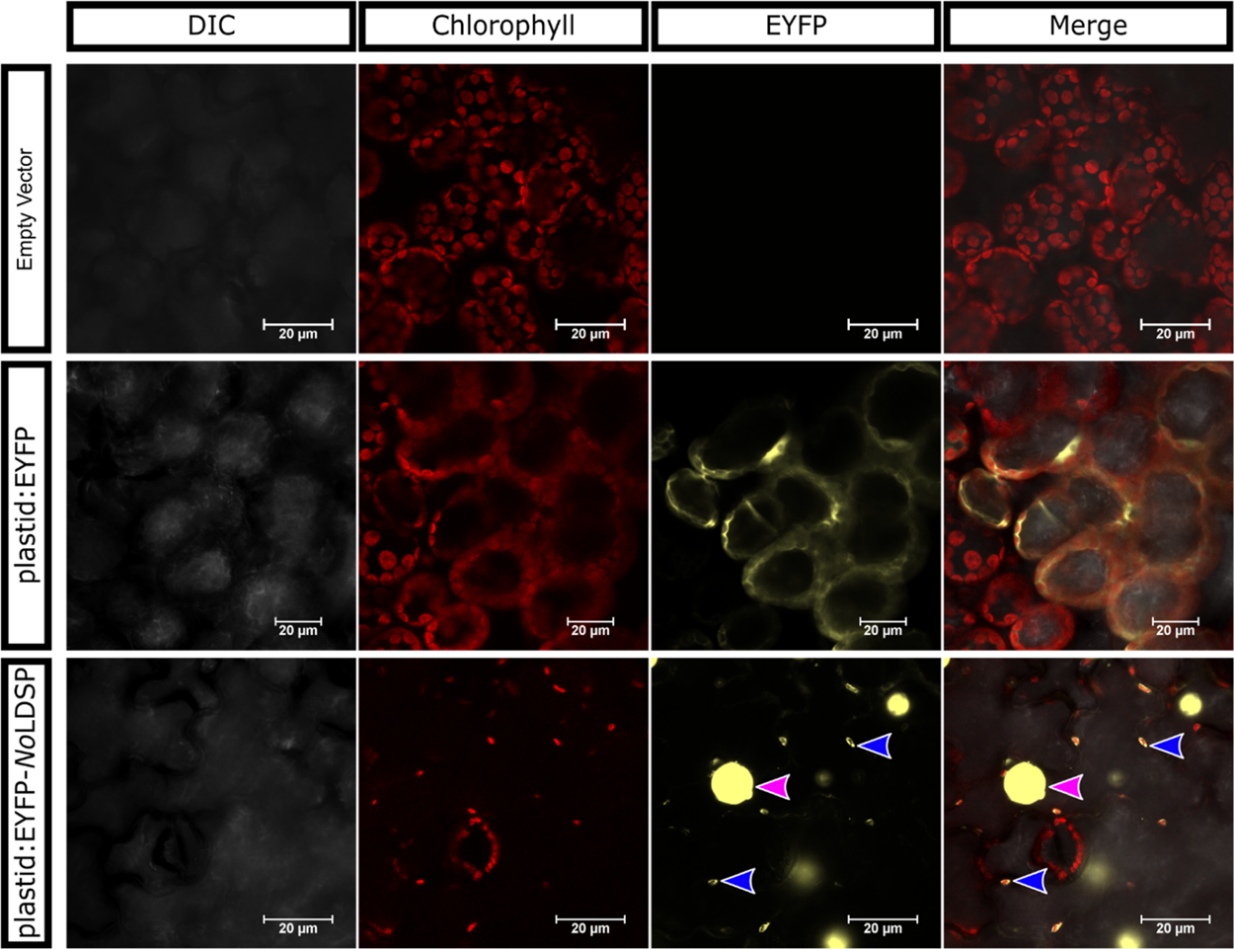
Confocal microscopy of plastid-targeted EYFP and EYFP-*No*LDSP to compare membrane localization. EYFP fluorescence and chlorophyll autofluorescence were measured with excitation:emission wavelengths of 513.9 nm:585 nm and 561 nm:700 nm, respectively. Blue arrows point to EYFP punctate seen in chloroplasts; pink arrows point to EYFP aggregating at cytosolic lipid droplets still seen being formed.

First, confocal microscopy was performed on *Agrobacterium*-infiltrated *N. benthamiana* containing constructs for an empty vector (EV), cyt:EYFP, cyt:EYFP-*No*LDSP, plast:EYFP, or plast:EYFP-*No*LDSP. It was previously reported and confirmed with Nile Red staining that *NoLDSP* overexpression induces lipid droplet formation.^29^ This is confirmed here, where the cyt:EYFP-*No*LDSP fusion can be seen aggregating to cytosolic droplets (Figure S2). In plants expressing the genes for plast:EYFP-*No*LDSP, the fusion protein appears to aggregate along the plastid envelopes as well as forming a distinct punctate pattern within chloroplasts (Figure 4). This suggested plast:EYFP-*No*LDSP localization to multiple membranes within chloroplasts, including plastoglobule-like structures. In the plast:EYFP-*No*LDSP lines, likely cytosolic lipid droplet structures are also seen, suggesting that *No*LDSP may still be forming lipid droplets before the fusion protein can be transported to plastids. Future engineering of chloroplast genomes for plastidial expression of LDSP fusion genes may prevent cytosolic localization and induction of lipid droplet formation.

**Figure 5:**
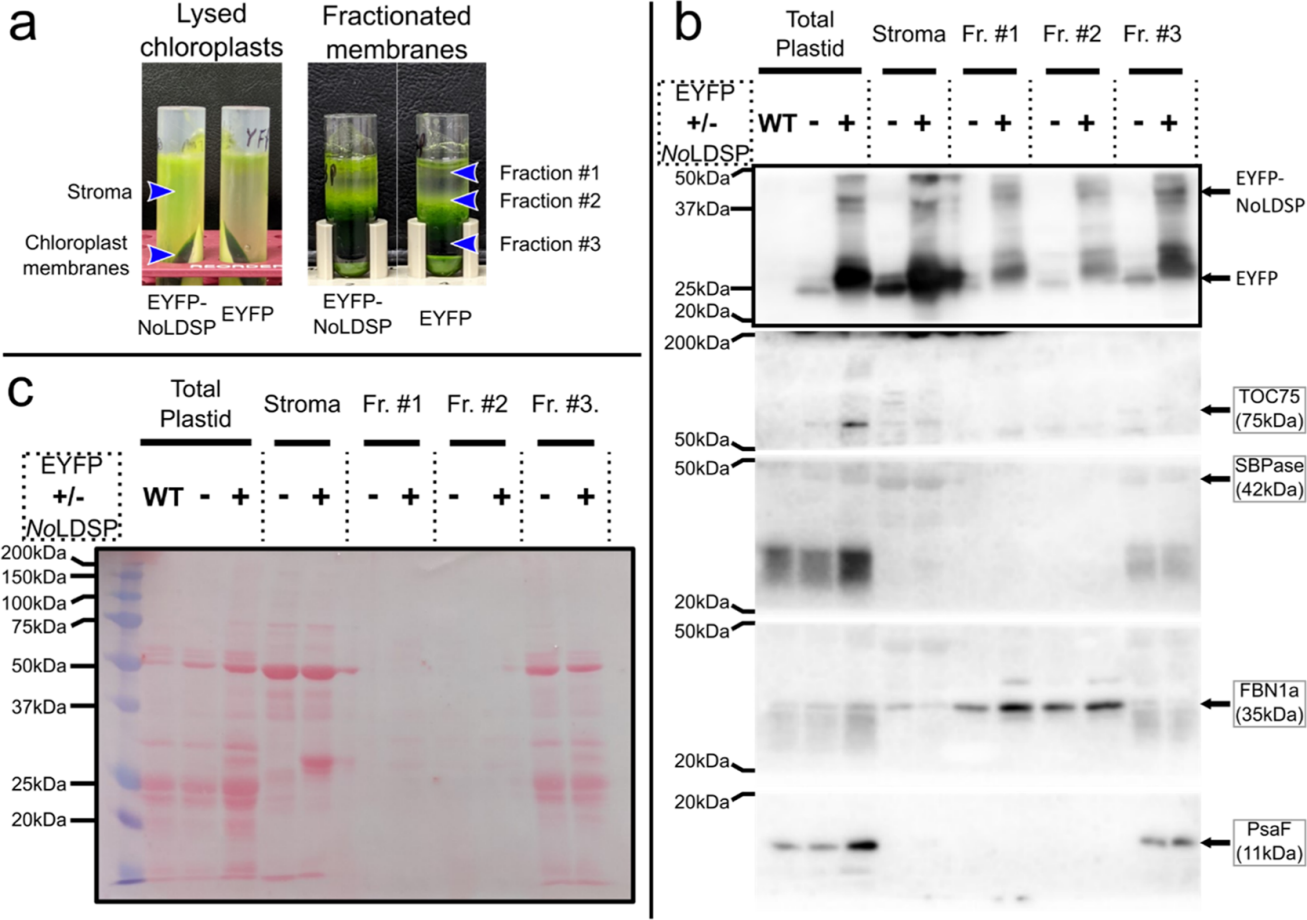
Western blots to determine membrane localization of the plastid target, EYFP-*No*LDSP fusion proteins. Chloroplast fractions (a) are labeled, where the different samples were taken. Western blots using antibodies specific to proteins from each membrane fraction (b) are labeled with the expected protein mass and the ladder markers where the membrane was cut for antibody application. The intact membrane was stained with Ponceau S dye (c) prior to cutting fragments for antibody visualization. Each lane is indicated as uninfiltrated, wild-type plants (WT) or the presence of EYFP with (+) or without (−) fusion to *No*LDSP. Arrows indicate bands at the expected sizes of each protein of interest. In (b), the membrane fragment cut at 50 kDa and 20 kDa was first visualized with anti-FBN1a, followed by anti-SBPase. To analyze EYFP and EYFP-*No*LDSP, the 50 kDa–20 kDa membrane was then visualized using an anti-GFP antibody.

Previous studies have demonstrated the ability to isolate chloroplasts, separate the stroma, and fractionate plastidial membranes to study proteins associated with each membrane type.^50,51^ To determine localization of plast:EYFP-*No*LDSP fusion proteins within chloroplasts, further analysis was performed here through chloroplast isolation and membrane fractionation with whole-plant, vacuum-infiltrated lines expressing plast:*EYFP* or plast:*EYFP-NoLDSP*. Chloroplasts were isolated and lysed, and membranes were separated from the stroma into three fractions (Figure 5a and S3a). The plast:EFYP-*No*LDSP fusion proteins were found associating with the stroma and all three membrane fractions, determined by the anti-GFP antibody (Figures 5b and S3b). Also seen in each fraction is what may be a cleavage product of the plast:EYFP-*No*LDSP fusion protein closer to the size of EYFP, although further analysis would be needed to confirm.

The stromal fraction was confirmed by application of the antibody for sedoheptulose-1,7-bisphosphatase (anti-SBPase) (Figure 5b and S3b). Fractions #1 and #3 were confirmed to contain plastoglobules and thylakoids, respectively, as determined by antibodies for fibrillin 1a (anti-FBN1a) and a subunit of photosystem I (anti-PsaF) (Figure 5b and S3b). Fraction #2 was inconsistent between experiments when visualized with antibodies for the 75 kDa subunit of the Translocon of the Outer Chloroplast (anti-TOC75), a protein associated with the outer membrane of the chloroplast envelope. TOC75 was not detected in Figure 5b but is detected in Figure S3b, suggesting that this fraction contains chloroplast envelopes. In support of these findings, a previous study visualized a similar fraction with anti-TOC75 and determined it to be predominantly chloroplast envelopes.^50^ Plastidial targeting of *No*LDSP fusion proteins, therefore, enables non-specific, membrane scaffolding of proteins and pathways throughout the chloroplast.

### Incorporating Alternative Contributions for the MVA Pathway

To determine if squalene yields could be improved by introducing alternative contributions to the MVA pathway, further gene screenings were performed. While archaea rely on the MVA pathway, most lack the final two enzymes of the classical pathway to form IDP.^52^ In the classical MVA pathway, phosphomevalonate (MVAP) is phosphorylated by MVAP kinase to form mevalonate diphosphate (MVADP), followed by decarboxylation by MVADP decarboxylase to form IDP. In this alternative pathway (Figure 1), MVAP is first decarboxylated by phosphomevalonate decarboxylase (MPD) to form isopentenyl phosphate (IP), which is then phosphorylated to IDP with an IP kinase (IPK).

Recently, cytosolic pools of IP were found and IPK orthologs were identified in plants,^25^ suggesting a role of IPK in regulation of IDP concentrations. While no MPD orthologs have been discovered in plants, co-expression of the MPD gene from the bacterium *R. castenholzii* (*RcMPD*) with the *A. thaliana* IPK gene (*AtIPK*) showed increased production rates of terpenoids in the *Nicotiana tabacum* transient expression system.^26^ This alternative pathway toward IDP was tested here to determine compatibility with the lipid droplet scaffolding strategy and to possibly increase squalene yields further.

In a separate approach to increase yields of cytosolic terpenoid pathways, overexpression of a biotin carboxyl carrier protein gene from *A. thaliana* (*AtBCCP1*) was investigated. It was previously shown that overexpression of a second BCCP isoform, *AtBCCP2*, resulted in accumulation of non-biotinylated BCCP and a reduction in fatty acid levels.^63^ It was suggested that non-biotinylated BCCP may be incorporated to form an inactive acetyl-CoA carboxylase complex, reducing conversion of acetyl-CoA to malonyl-CoA, the committed step towards plastidial *de novo* fatty acid biosynthesis.^63^ Building upon this work, a separate study overexpressed either *AtBCCP1* or *AtBCCP2* in tandem with terpenoid pathways which led to increased yields of cytosolic terpenoids, presumably by allowing more acetyl-CoA to enter the MVA pathway.^22^ In attempts to further increase cytosolic squalene yields, *AtBCCP1* overexpression was included here in combination with strategies developed for cytosolic squalene production (Figure 1).

These genes were co-expressed with the cytosolic squalene pathway, *ElHMGR*^159-582^ and cyt:*AtFDPS*, both with and without lipid droplet scaffolding for comparison (Figure 3b). The addition of *At*IPK alone or *At*IPK and *Rc*MPD did not increase squalene yields. When combined with the lipid droplet scaffold, the addition of both *At*IPK and *Rc*MPD significantly reduced squalene yields. In this work, *At*IPK and *Rc*MPD were tested in an entirely transiently expressed system to synthesize the non-volatile squalene. The initial work was performed by transiently expressing the pathway for the volatile sesquiterpene santalene in a transgenic *RcMPD* overexpression line of *Nicotiana tabacum*.^26^ The rates of santalene emission were increased with overexpression of *RcMPD* and *AtIPK*, but overall yields were not measured. While the use of these enzymes may increase the rates of terpenoid production, they did not increase overall squalene yields in this study. There was also no increase in squalene yields when *At*BCCP1 was added to transient expression systems here. The initial work characterizing the role of *At*BCCP1 in cytosolic terpene production showed increased yields of the sesquiterpene bisabolol when overexpressing bisabolol synthase, *HMGR*, and *BCCP1*. While *AtBCCP1* overexpression, as well as *AtIPK* and *RcMPD*, did not increase squalene yields here, this may be due to limitations in storage capacity rather than enzyme activities. The increase in squalene yields when lipid droplets are co-produced (Figure 3b) demonstrates metabolic capability to produce more squalene but may require greater storage capacity.

### Investigating How Expression of Squalene Pathways Affects Photosynthesis

**Figure 6:**
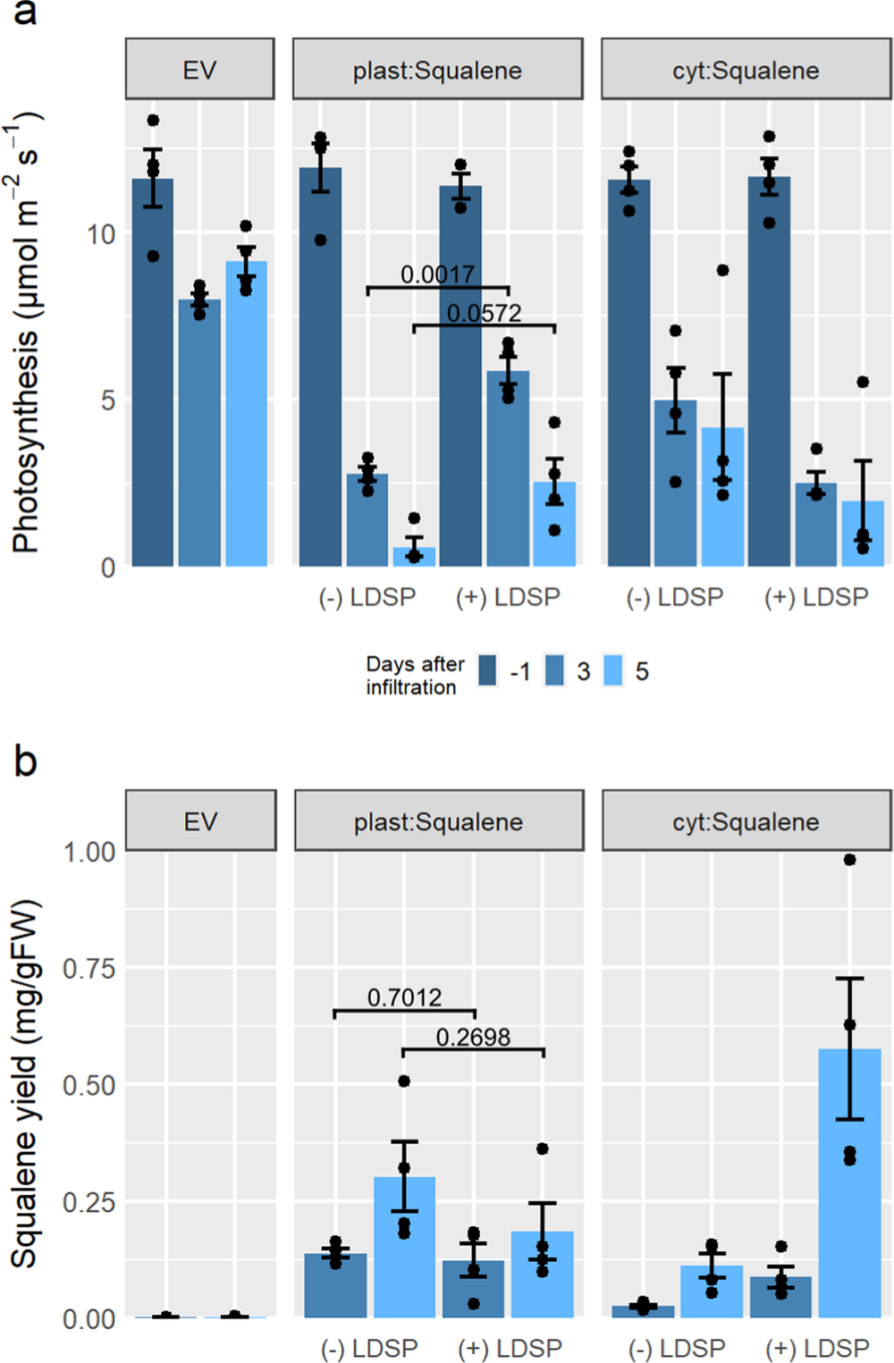
Comparison of photosynthesis and squalene biosynthesis in leaves transiently expressing cytosolic and plastid-targeted squalene pathways. Photosynthesis data (a) and squalene yields (b) for each set of plants compared between the EV, plastid squalene pathways (plast:Squalene) with (+LDSP) and without (−LDSP) membrane scaffolding, and cytosolic pathways (cyt:Squalene) with and without lipid droplet scaffolding. Black circles show individual data points, and bars represent means ± standard error. *n* = 4 plants per treatment. Individual *t*-test statistical comparisons between means are shown by brackets and the indicated p-value.

Engineered biosynthetic pathways and accumulation of products in plants have been reported to hinder overall productivity and result in negative phenotypes such as stunted growth and reduced photosynthesis.^10−12,30,31^ To evaluate possible effects of these engineered pathways on native physiology, a series of gas-exchange experiments were performed under various squalene-producing conditions. Although pathways were developed here using transient expression in vacuum-infiltrated *N. benthamiana*, these experiments provide insight into how they may affect stably transformed crops. Four squalene strategies were evaluated for their effect on photosynthesis as an indicator for influences on plant physiology: (i) cyt:SQ_(−) LDSP_ (cyt:*El*HMGR + cyt:*At*FDPS + cyt:*Ma*SQS CΔ17), (ii) cyt:SQ_(+) LDSP_ (*At*WRI1 + cyt:*El*HMGR + cyt:SQS-LDSP-FDPS), (iii) plast:SQ_(−) LDSP_ (plast:*Cf*DXS + plast:*At*FDPS + plast:*Ma*SQS CΔ17), and (iv) plast:SQ_(+) LDSP_ (plast:*Cf*DXS + plast:SQS-LDSP-FDPS). Each strategy reduced overall photosynthesis on both days 3 and 5, compared to pre-infiltration measurements (Figure 6a). Plants infiltrated with 200 μM acetosyringone in water or with *Agrobacterium* containing an empty pEAQ-*HT* vector showed no significant differences in photosynthesis and squalene yields compared to uninfiltrated plants (Figure S4). This indicates that effects on leaf physiology reported here were due to expression of genes involved in the various squalene pathways studied.

Both cytosolic strategies, with and without lipid droplet scaffolding, reduced photosynthesis on day 3 and then further on day 5 (Figure 6a). Comparing the soluble pathways cyt:SQ_(−) LDSP_ and plast:SQ_(−) LDSP_, the cyt:SQ_(−) LDSP_ pathway had 81% higher photosynthetic rates than plast:SQ_(−) LDSP_ on day 3 and 631% higher photosynthetic rates on day 5. Cytosolic lipid droplet scaffolding (cyt:SQ_(+) LDSP_) caused a greater reduction in photosynthesis than without, which is likely due to overexpression of *AtWRI1* as previous studies have shown that WRI1 induces severe downregulation of gene expression for proteins involved in the photosynthetic apparatus.^45^ Targeting the lipid droplet scaffold to plastids, however, moderated some of the negative effects on photosynthesis. Compared to the plast:SQ_(−) LDSP_ pathway on day 3, the plast:SQ_(+) LDSP_-infiltrated plants had 112% higher levels of photosynthesis, and on day 5, plast:SQ_(+) LDSP_ had 345% higher levels of photosynthesis than plast:SQ_(−) LDSP_. These data demonstrate that scaffolding of the plastidial, squalene biosynthetic pathway partially ameliorates the negative effects of squalene biosynthesis on photosynthesis.

The differences in photosynthesis seen in response to the expression of different squalene pathways were not due to variations in intercellular [CO_2_] resulting from differences in stomatal conductance (data not shown). We analyzed *A*/*C*_i_ curves [photosynthetic rate (*A*) plotted against intercellular CO_2_ concentration, *C*_i_] to understand what biochemical properties of photosynthesis are affected by squalene production (Figure S5). Changes in rubisco activity (*V*_c,max_) and ribulose 1,5-bisphosphate regeneration (or the rate of electron transport, *J*) followed similar trends and degrees of change as seen for photosynthesis in leaves expressing different squalene pathways (Table S2). This shows that the negative effects on photosynthesis in leaves expressing squalene pathways were mainly due to reduced rubisco activity and that targeting the LDSP scaffolds to plastids can moderate some of the negative effects on rubisco and photosynthesis. By comparing photosynthesis rates (Figure 6a) with squalene production (Figure 6b), it is clear that the increase in photosynthesis in plast:SQ_(+) LDSP_ was due to scaffolding of the plastidial squalene biosynthetic pathway and not due to a decrease in squalene production. The reason for how squalene negatively affects rubisco and how scaffolding of the plastidial squalene biosynthetic pathway helps alleviate the negative effects on rubisco and photosynthesis can only be speculated at this time. It may be that squalene accumulation in plastids has a direct inhibitory effect on rubisco. Studies have demonstrated that squalene accumulation can form aggregates and interfere with native membranes.^53,54^ Compared to the soluble plastid pathway, the LDSP scaffolding in plastids may distribute the accumulation of squalene more broadly throughout various membranes and reduce disruption of protein organization on thylakoid membranes.

While squalene biosynthesis was detected in leaves expressing all squalene pathways tested, the largest increase was seen with the cytosolic strategy with lipid droplet scaffolding (Figure 6b). In comparison, squalene production by the plastidial pathways was less than half that of the cytosolic pathway with lipid droplet scaffolding. Additionally, when determining the amount of fixed carbon utilized in the engineered squalene pathways, plast:SQ_(−) LDSP_ presented the highest conversion of 1.97% of fixed carbon toward squalene (Table 1), while all other strategies utilized less than 1% of fixed carbon. Compared to the microalgae *B. braunii*, in which upward of 45% of photosynthetic carbon is naturally directed toward terpenoid biosynthesis,^55^ there may be significant capacity to increase carbon partitioning toward squalene in these engineered systems.

**Table 1:** Summary of changes in squalene yields and photosynthesis when scaffolding squalene biosynthesis using *No*LDSP^a^

treatment	day after infiltration	squalene yield (mg/gFW)	difference in squalene yield with LDSP	difference in photosynthesis with LDSP	squalene production (μmol/m^2^)	photosynthetic carbon fixed (μmol/m^2^)	% offixed carbon to squalene
empty vector	day 3	9.49 × 10^–4^			1.30	3.45 × 10^5^	0.00%
empty vector	day 5	1.66 × 10^–3^			2.69	3.94 × 10^5^	0.00%
plast:SQ(−)LDSP	day 3	1.38 × 10^–1^	–11.2%	+112%	1.82 × 10^2^	1.19 × 10^5^	0.15%
plast:SQ(+)LDSP		1.23 × 10^–1^			1.78 × 10^2^	2.52 × 10^5^	0.07%
plast:SQ(−)LDSP	day 5	3.02 × 10^–1^	–38.9%	+345%	4.84 × 10^2^	2.46 × 10^5^	1.97%
plast:SQ(+)LDSP		1.84 × 10^–1^			3.14 × 10^2^	1.10 × 10^5^	0.29%
cyt:SQ(−)LDSP	day 3	6.28 × 10^–2^	+67.6%	–49.8%	8.33 × 10^2^	2.15 × 10^5^	0.04%
cyt:SQ(+)LDSP		1.05 × 10^–1^			1.44 × 10^2^	1.08 × 10^5^	0.13%
cyt:SQ(−)LDSP	day 5	1.11 × 10^–1^	+318%	–52.9%	1.48 × 10^2^	1.80 × 10^5^	0.08%
cyt:SQ(+)LDSP		5.75 × 10^–1^			7.76 × 10^2^	8.47 × 10^5^	0.92%

a Changes are summarized for the plastid (plast:SQ)- or cytosol (cyt:SQ)-targeted pathways. Squalene production and photosynthetic carbon fixed during a 12 h photoperiod were calculated in terms of μmol of carbon fixed per unit surface area (m^–2^) of the leaf sampled, and the ratio was used to determine the percentage of photosynthetic fixed carbon converted to squalene (% of fixed carbon to squalene). Values represent means obtained from *n* = 4 plants per treatment.

In conclusion, optimizing key steps in squalene biosynthesis and compartmentalizing the pathway at cytosolic lipid droplets or within plastids and plastid membranes effectively improve production of squalene within plant systems. These strategies provide a platform which can be expanded to produce compounds directly formed from squalene, such as ambrein, or other squalene-derived triterpenoids, sterols, and related bioproducts with important industrial applications. Combining these pathways with alternative precursor contributors without seeing increased yields suggests that there may be a need to further increase storage capacity. Additionally, there is significant photosynthetic capacity to direct more fixed carbon to products. Both may be improved by further engineering of the lipid droplet architecture or membrane scaffolding to increase overall storage capacity. Experiments here were performed using *Agrobacterium*-mediated transient expression, which may inform future work to generate stable transformants.

## Methods

### Genes Synthesized and Cloned

Genes used in this study are listed in Table S1. Genes were either cloned from cDNA from the native host or synthesized as gene fragments from Integrated DNA Technologies (IDT) or Twist Bioscience. The IDT Codon Optimization Tool was used for genes codon-optimized for expression in *N. benthamiana*. Genes were initially inserted into the pJET 1.2/blunt vector using a CloneJET PCR Cloning Kit (ThermoFisher Scientific). For transient expression experiments, genes were amplified with Phusion High-Fidelity DNA Polymerase (New England Biolabs) and then inserted into the pEAQ-*HT* vector^56,57^ (digested with XhoI and NruI restriction enzymes) using an In-Fusion HD Cloning Kit (Takara Bio). The pEAQ-*HT* vector (Genbank GQ497234.1) utilizes an enhanced expression platform using the cauliflower mosaic virus 35S promoter, 5′ and 3′ untranslated regions from the cowpea mosaic virus, and the nopaline synthase terminator. Additionally, this vector co-expresses the RNA-silencing suppressor P19 gene using the 35S promoter and terminator from the cauliflower mosaic virus. All genes following pJET 1.2/blunt and pEAQ-*HT* cloning were confirmed through Sanger sequencing provided by Psomagen, Inc. *Agrobacterium tumefaciens* LBA4404 cells were transformed with pEAQ-*HT* plasmids containing the gene of interest via electroporation and then plated on LB media with 50 μg/mL of kanamycin and 25 μg/mL of rifampicin for selection. Transformed *Agrobacterium* cells were cultured overnight and flash-frozen in 20% glycerol for storage until they were needed for transient expression.

### *Agrobacterium*-Mediated Transient Expression, Compound Extraction, and Measurement

*Agrobacterium*-mediated, transient expression experiments were performed similar to previously described methods.^29^ Transformed *Agrobacterium* cells from 20% glycerol stocks were cultured in 5mL LB with 50 μg/mL kanamycin and 25 μg/mL rifampicin for 20 h at 28 °C. These starter cultures were used to inoculate 25 mL of LB with 50 μg/mL kanamycin, also cultured for 20 h at 28 °C. Cultures were then centrifuged at 4000 *g* for 10 min, decanted, and then re-suspended in 10 mL of water. This was repeated two more times for a total of three washes and finally re-suspended in 10 mL of water with 200 μM of acetosyringone, which was diluted to an OD_600_ of 0.8 or 1.0 with 200 μM acetosyringone in water. Cultures for each set of DXS experiments were diluted to OD_600_ of 0.8, while cultures for each set of squalene pathway optimization experiments were diluted to OD_600_ of 1.0. Following dilution, cultures were shaken at 28 °C for 1–2 h before infiltration. Cultures for co-infiltration were mixed in equal proportions and then syringe-infiltrated into three leaves of three *N. benthamiana* plants (4–5 weeks old) for a total of nine replicates of each combination in each experiment. Infiltrated plants were left under growth conditions for 5 days before extraction of compounds. *N. benthamiana* plants used for these infiltrations were grown at 23–25 °C with a 12 h photoperiod at 150 μmol m^–2^ s^–1^.

For casbene extractions, 2–15 mm leaf discs were cut out and placed in a vial with hexane containing 20ng/μL ledol. Samples were left shaking overnight at room temperature and centrifuged at 525*g* for 20 min to pellet plant debris, and the supernatant was transferred to fresh amber GC vials for analysis. For squalene extractions, 2–15 mm leaf discs were cut out and placed in 2 mL screw cap vials containing 0.1 mm glass beads and 1–3 mm tungsten carbide beads and then flash-frozen in liquid nitrogen and stored in −80 °C until extraction. Frozen leaf tissue samples were ground using a Qiagen TissueLyser at 30 rotations s^–1^ for 1.5 min twice, and 600 μL of hexane containing 50 ng/μL *n*-hexacosane was added and samples were vortexed; then, samples were shaken for 2 h at room temperature. 300 μL of water was added to aid with separation, samples were centrifuged at 16 000*g* for 5 min, and the organic layer was transferred to amber GC vials for analysis.

Hexane extracts were analyzed via gas chromatography–flame ionization detection (GC-FID) on an Agilent 7890A and compared to retention times of a squalene standard. Peak areas for the internal standard, hexacosane, and squalene were extracted for comparison. To determine relative yields of squalene, or casbene, peak areas of squalene were divided by hexacosane, or ledol, peak areas for a ratio for comparison. For quantification of squalene, the fresh leaf tissue was weighed prior to extraction and a squalene, GC-FID calibration curve was created to determine yields. Squalene peak areas were normalized to the mean of hexacosane areas across samples, and squalene was quantified based on the calibration curve.

### Plastid Fractionation and Western Blots

Plastids from *Agrobacterium*-infiltrated leaves were extracted and fractionated similar to previously described methods.^51^ Whole *N. benthamiana* plants were vacuum-infiltrated with *Agrobacterium*-harboring pEAQ-*HT* vectors which contain either a plast:EYFP gene or a plast:EYFP-*No*LDSP fusion gene. Leaves from 15 full plants from each condition were blended in an isolation buffer (330 mM sorbitol, 20 mM (3-(N-morpholino)propanesulfonic acid) pH 7.6, 13 mM Tris–HCl, 3 mM MgCl_2_, 0.1% (w/v) BSA, 5 mM ascorbic acid, 5 mM reduced cysteine, and a protease inhibitor mixture containing 74 μM antipain, 130 μM bestatin, 16.5 μM chymostatin, 56 μM E64, 2.3 μM leupeptin, 37 μM phosphoramidon, 209 μM 4-(2-aminoethyl)benzenesulfonyl fluoride hydrochloride, 0.5 μM aprotinin, 50 mM NaF, 25 mM b-glycerophosphate, 1 mM Na-orthovanadate, and 10 mM Na-pyrophosphate) and then filtered through Miracloth. Samples were centrifuged for 5 min at 1500*g* at 4 °C to pellet chloroplasts; then, pellets were washed twice with a washing buffer [50 mM *N*-(2-hydroxyethyl)piperazine-*N*′-ethanesulfonic acid (HEPES) pH 7.6, 5 mM ascorbic acid, 5 mM reduced cysteine, 330 mM sorbitol, and the protease inhibitor mixture] and centrifuged again as described above. To break open chloroplasts, washed samples were re-suspended and incubated for 30 min on ice in an osmotic shock buffer (10 mM Tricine pH 7.9, 1 mM ethylenediaminetetraacetic acid, 0.6 M sucrose, and the protease inhibitor mixture). Lysed chloroplasts were centrifuged for 1 h at 100,000*g* to separate the chloroplast stroma from membranes. The membrane pellet was resuspended in 2 mL of 48% sucrose in HE buffer (50 mM HEPES pH 7.9 and 2 mM EDTA); 1mL was transferred to an ultra-centrifuge tube where 800 μL 5% sucrose in HE buffer was carefully overlaid, and the gradient was centrifuged for 2 h at 100 000*g*. The top, yellow layer consisting of plastoglobules, the second, yellow layer consisting of plastid envelopes, and the bottom, green layer consisting of thylakoids were removed for analysis (Figures 5a and S3a).

For each membrane layer, total protein was quantified using a Pierce BCA Protein Assay Kit (Thermo Scientific) and 5 μg of total protein was added to Laemmli sample buffer before boiling for 10 min. Each boiled sample was run on a 12% sodium dodecyl sulfate (SDS) gel with 4% SDS stacking gel to separate proteins; then, proteins were transferred to a nitrocellulose membrane (Amersham Protran), and total proteins were stained and visualized with incubation in Ponceau S dye (Figures 5c and S3c). The dye was removed, and the membrane was incubated in a blocking buffer [5% condensed milk in Tris-buffered saline (TBS)] at room temperature for 1 h. Membranes were washed in TBS with 0.1% Tween 20 (TBS-T), cut at the specified molecular weight markers, and then incubated at 4 °C overnight with the appropriate antibody. The antibodies anti-SBPase, anti-TOC75, anti-PsaF, and anti-FBN1a were used to identify fractions for the stroma, plastid envelopes, thylakoids, and plastoglobules, respectively. Membranes were washed with TBS-T, incubated with the secondary, polyclonal anti-rabbit horseradish peroxidase antibody for 2 h at room temperature, washed again, and then imaged using enhanced chemiluminescence. For membranes with EYFP, the secondary antibody was stripped and washed, and the anti-GFP antibody was applied and imaged as above, starting with the blocking buffer.

### Gas-Exchange Measurements

*N. benthamiana* plants for gas-exchange measurements were grown from seeds in Suremix (Michigan Grower Products) in 5″ pots. Plants were grown under a 12 h photoperiod, a light intensity of 400 μmol m^–2^ s^–1^, day/night temperatures of 25°C/20 °C, and 60% humidity. Plants were kept in a growth chamber (Big-Foot, BioChambers) and fertilized using 1/2-strength Hoagland’s solution.^58^ 5-week-old plants were infiltrated with *Agrobacterium* harboring vectors for the indicated squalene pathways and controls at an OD_600_ of 0.8 as described above, except using vacuum instead of syringe infiltration. For vacuum infiltration, whole plants were submerged in the respective *Agrobacterium* cultures and placed under vacuum for 3–4 min; then, vacuum was quickly released to infiltrate leaves. The vacuum and release was repeated once to ensure full infiltration of leaves.

Gas-exchange measurements described below were performed the day before infiltration of leaves and on the 3rd and 5th days after infiltration. For each plant, gas-exchange measurements were performed on the third fully expanded leaf counting from the top of the canopy. This leaf was tagged, and the same leaf was measured consecutively prior to infiltration and on the 3rd and 5th days after infiltration. For squalene measurements, on day 3, 4–15 mm leaf discs were collected from the leaf immediately above the leaf being used for gas-exchange measurements. On day 5, leaf discs were collected from the leaf being measured following measurements at the end of the day. Squalene was extracted and measured as described above.

Photosynthetic rates (*A*), the operational efficiency of photosystem II in light-adapted leaves (Φ_PSII_), and stomatal conductance (*g*_sw_) were measured simultaneously with the aid of an LI-6800 portable gas-exchange system (LI-COR Biosciences, Lincoln, NE) connected to a Multiphase Flash fluorometer (6800-01A). The environmental conditions inside the LI-6800 leaf chamber were set to match daytime growth chamber conditions: a light intensity of 400 μmol m^–2^ s^–1^ (50% blue light and 50% red light), a temperature of 25 °C, a CO_2_ concentration of 400 μmol mol^–1^, and a water vapor content of 22 mmol mol^–1^. First, the leaf was inserted into the leaf chamber and allowed to equilibrate for 30 min under the above conditions. A measurement was logged after photosynthesis reached the steady state at the end of this equilibration period. Next, the light intensity inside the leaf chamber was then increased to 1000 μmol m^–2^ s^–1^, and the leaf was held under this saturating light condition until photosynthesis reached the steady state. The response of photosynthesis to CO_2_ was determined by measuring the photosynthetic rates at varying [CO_2_]. [CO_2_] was set to change from low to high (50, 100, 150, 200, 300, 350, 400, 450, 500, 550, 600, 7000, 800, 1000, 1300, 1500 μmol mol^–1^), and the leaf was allowed to equilibrate for 2–3 min at each CO_2_ concentration before a measurement was logged. These data were used to plot *A*/*C*_i_ curves. To determine the biochemical capacities underlying photosynthesis, namely, maximum carboxylation rate (*V*_c,max_), maximum rate of electron transport (*J*), and triose phosphate utilization rate (TPU), A/Ci curves were fitted by the Farquhar–von Caemmerer–Berry biochemical model of photosynthesis,^59,60^ using the *A*/*C*_i_ curve fitting utility version 2.9 for tobacco.^60−62^

## Supporting Information Available

Genes used in this study and their associated accession numbers, analysis of photosynthesis response to CO_2_ in leaves expressing plastid-targeted and cytosolic squalene pathways with and without *No*LDSP scaffolding, additional boxplots comparing soluble and *No*LDSP scaffolding pathways in the cytosol and plastids, confocal microscopy comparing cytosolic EYFP and EYFP-*No*LDSP, additional plastid fractionation and western blots demonstrating EYFP-*No*LDSP localization in chloroplast membranes, comparison of the effects of transient expression of plastid-targeted squalene pathways in leaves and subsequent effects on photosynthesis compared to controls, and *A*/*C*_*i*_ curves comparing plastid-targeted and cytosolic squalene pathways with and without *No*LDSP scaffolding (PDF)

## Abbreviations

SQS: squalene synthase
FDPS: farnesyl diphosphate synthase
LDSP: lipid droplet surface protein
DXS: 1-deoxy-d-xylulose-5-phosphate synthase
HMGR: 3-hydroxy-3-methylglutaryl-CoA reductase
GAP: glyceraldehyde 3-phosphate
Ru5P: ribulose 5-phosphate
IDP: isopentenyl diphosphate
DMADP: dimethylallyl diphosphate
GGDPS: geranylgeranyl diphosphate synthase
CasS: casbene synthase
IDI: isopentenyl diphosphate isomerase
WRI1: WRINKLED1
MPD: phosphomevalonate decarboxylase
IPK: isopentenyl phosphate kinase
BCCP1: biotin carboxyl carrier protein
SBPase: sedoheptulose-1,7-bisphosphatase
FBN1a: fibrillin 1a
PsaF: photosystem I subunit F
TOC75: Translocon of the Outer Chloroplast
*C*_i_: intercellular CO_2_ concentration
*A*: photosynthetic rate
*V*_c,max_: rubisco activity
*J*: electron transport rate
Φ_PSII_: operational efficiency of photosystem II
*g*_sw_: stomatal conductance
TPU: triose phosphate utilization rate
