# Supplementary material for: Pathway Engineering, Re-targeting, and Synthetic Scaffolding Improve the Production of Squalene in Plants
Source: ACS Synth Biol. 2022 May 13;11(6):2121–33. doi: 10.1021/acssynbio.2c00051 (PMC9208017; doi:10.1021/acssynbio.2c00051)
Supplement: Supplementary file 1 — sb2c00051_si_001.pdf [file sb2c00051_si_001.pdf]

Pathway engineering, re-targeting, and synthetic scaffolding improves production of squalene in plants

Jacob D. Bibik[1,2,3], Sarathi M. Weraduwage[3,4], Aparajita Banerjee[2,3], Ka'shawn Robertson[2], Roberto Espinoza-Corral[3,5], Thomas D. Sharkey[3,4,5], Peter K. Lundquist[3,5], and Björn R. Hamberger[1,2,3]*

[1]Cell and Molecular Biology Program, Michigan State University, East Lansing, Michigan, United States of America

[2]DOE Great Lakes Bioenergy Research Center, Michigan State University, East Lansing, Michigan, United States of America

[3]Department of Biochemistry and Molecular Biology, Michigan State University, East Lansing, Michigan, United States of America

[4]DOE Plant Research Laboratory, Michigan State University, East Lansing, Michigan, United States of America

[5]The Plant Resilience Institute, Michigan State University, East Lansing, Michigan, United States of America

*Corresponding author: hamberge@msu.edu

Table S1. Genes used in this study and their associated accession numbers.

Gene abbreviation	Organsim	Accession number
**DXS/nDXS variants**		
CfDXS	*Coleus forskohlii*	KP889115.1
PtDXS	*Populus trichocarpa*	EU693019.1
RibB G108S (nDXS)	*Escherichia coli*	WP_139956769.1
YajO (nDXS)	*Escherichia coli*	NP_414953.2
DXR	*Escherichia coli*	NP_414715.1
**FDP synthases**		
AtFDPS	*Arabidopsis thaliana*	NM_117823.4
PaFDPS	*Picea abies*	EU432049.1
GgFDPS	*Gallus gallus*	XM_015298647.1
**Squalene synthases**		
AhSQS	*Amaranthus hybridus*	AB691229.1
BbSQS	*Botryococcus braunii*	KT388100.1
ElSQS	*Euphorbia lathyris*	JQ694152.1
GlSQS	*Ganoderma lucidum*	DQ494674.1
MaSQS	*Mortierella alpina*	KT318395.1
ERG9	*Saccharomyces cerevisiae*	NP_012060.1
HolDISQS	*Haslea ostrearia*	AYV97147.1
**Other genes**		
CasS	*Daphne genkwa*	MZ485349.1
CfGGDPS	*Coleus forskohlii*	KP889114.1
AtWRI1 $^{1\text{-}397}$	*Arabidopsis thaliana*	AY254038.2
NoLDSP	*Nannochloropsis oceanica*	JQ268559.1
ElHMGR $^{159\text{-}582}$	*Euphorbia lathyris*	JQ694150.1
AtIPK	*Arabidopsis thaliana*	AY150412.1
RcMPD	*Roseiflexus castenholzii*	ABU57050.1
AtBCCP1	*Arabidopsis thaliana*	NM_121644.4

Table S2. Analysis of photosynthesis response to CO_2 in leaves expressing plastid targeted and cytosolic squalene pathways, with and without *No*LDSP scaffolding. A/C_i curves were fitted by the Farquhar-von Caemmerer-Berry biochemical model of photosynthesis using the following software: A/Ci curve fitting utility version 2.9 for tobacco[1–3], to determine how the biochemical capacities underlying photosynthesis: maximum carboxylation rate (V_{cmax}), maximum rate of electron transport (J), triose phosphate utilization rate (TPU), were affected in leaves expressing plastid targeted and cytosolic squalene pathways, with and without *No*LDSP scaffolding. Values represent means ± standard error. n = 4 plants per treatment.

	Before Infiltration			3 Days After Infiltration			5 Days After Infiltration		
Treatment	V_{cmax} (µmol m^{-2} s^{-1})	J (µmol m^{-2} s^{-1})	TPU (µmol m^{-2} s^{-1})	V_{cmax} (µmol m^{-2} s^{-1})	J (µmol m^{-2} s^{-1})	TPU (µmol m^{-2} s^{-1})	V_{cmax} (µmol m^{-2} s^{-1})	J (µmol m^{-2} s^{-1})	TPU (µmol m^{-2} s^{-1})
Empty Vector	77.4 ± 1.8	129.1 ± 4.7	7.9 ± 0.3	53.3 ± 4.9	91.0 ± 5.8	6.5 ± 0.3	55.3 ± 3.2	92.5 ± 5.6	6.2 ± 0.3
plast:SQ (-) LDSP	79.9 ± 2.3	120.2 ± 9.2	7.3 ± 0.5	18.4 ± 1.8	33.4 ± 2.4	2.3 ± 0.2	4.1 ± 0.9	10.0 ± 1.5	0.7 ± 0.1
plast:SQ (+) LDSP	76.1 ± 3.5	118.7 ± 7.8	7.6 ± 0.3	42.9 ± 3.8	68.1 ± 5.8	4.6 ± 0.4	15.8 ± 4.0	30.8 ± 6.3	2.2 ± 0.4
cyt:SQ (-) LDSP	80.9 ± 6.5	118.0 ± 6.5	7.3 ± 0.3	46.0 ± 6.2	72.3 ± 7.7	5.0 ± 0.6	30.1 ± 10.2	56.2 ± 14.2	4.0 ± 1.1
cyt:SQ (+) LDSP	82.3 ± 2.6	124.4 ± 6.9	7.8 ± 0.3	32.5 ± 5.9	57.0 ± 4.8	3.9 ± 0.3	16.8 ± 7.4	34.8 ± 10.9	2.5 ± 0.8

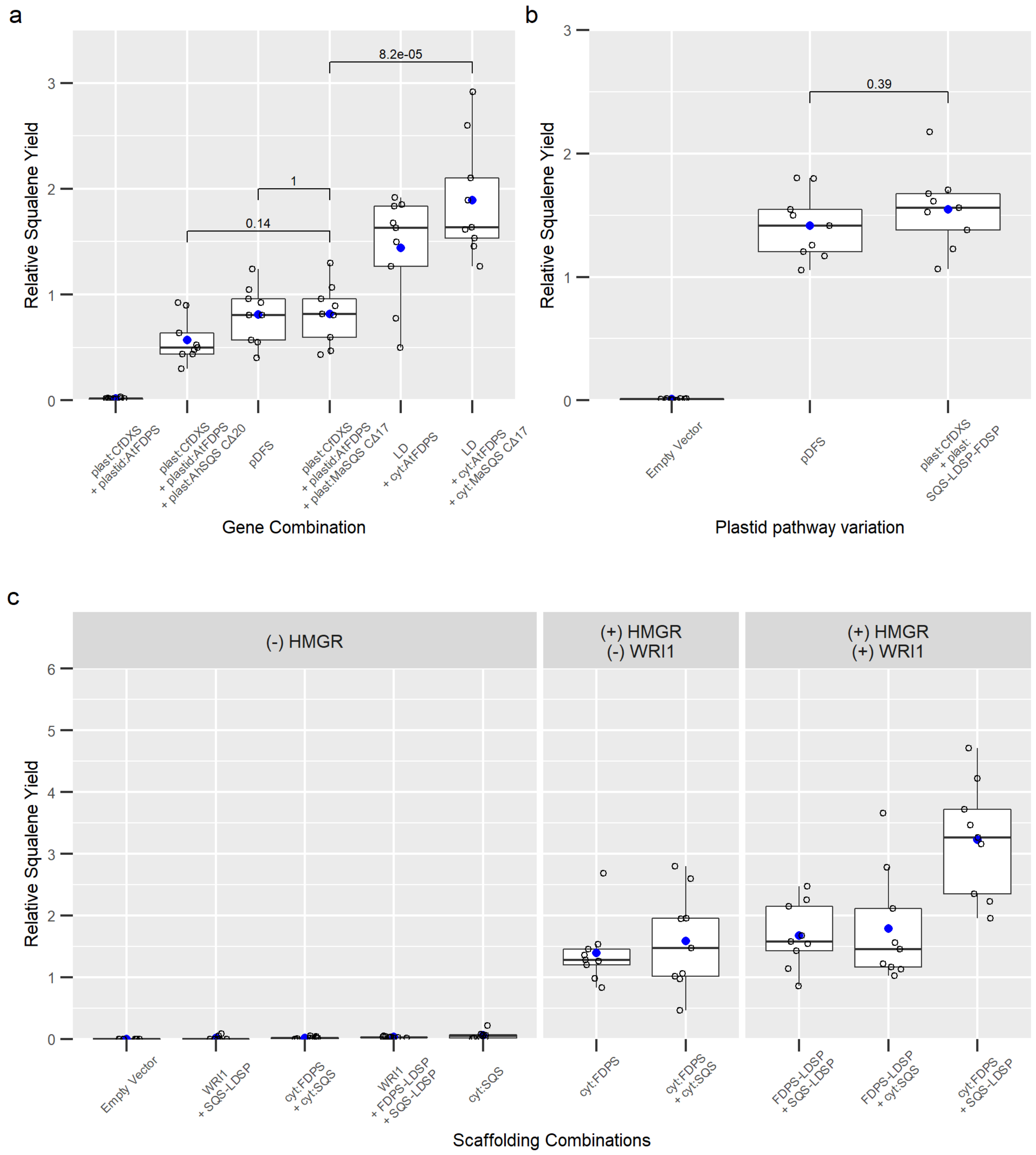

Figure S1. Additional boxplots comparing soluble and *No*LDSP scaffolding pathways in the cytosol and plastids. Panel (a) shows additional plastidial, SQS comparisons with pDFS (plast:*CfDXS*, plast:*AtFDPS*, and plast:*MaSQS CΔ17* separated by two LP4/2A hybrid linkers in pEAQ-*HT*) and the cytosolic co-production of lipid droplets, without scaffolding, and cyt:*ElHMGR*$^{159\text{-}582}$, cyt:*AtFDPS*, and cyt:*MaSQS CΔ17*. LD indicates co-expression with *AtWRI1*$^{1\text{-}397}$ and *NoLDSP*. Panel (b) shows comparisons between the pDFS vector and vectors used for plastid scaffolding in the photosynthesis experiments. Panel (c)

shows additional combinations of cytosolic lipid droplet scaffolding, including the initially tested *At*FDPS-*No*LDSP fusion variant. Each panel represents data from separate transient expression experiments. Open circles are individual data points, blue circles are mean value, and horizontal line within box represents the median value. The box shows the range from the lower 25th percentile to the upper 75th percentile. The upper and lower whiskers extend to the largest and smallest data point no further than 1.5x the inter-quartile range, with points lying outside the whiskers considered outliers. Paired statistical comparisons were performed by *t* test indicated by brackets with the corresponding p-values.

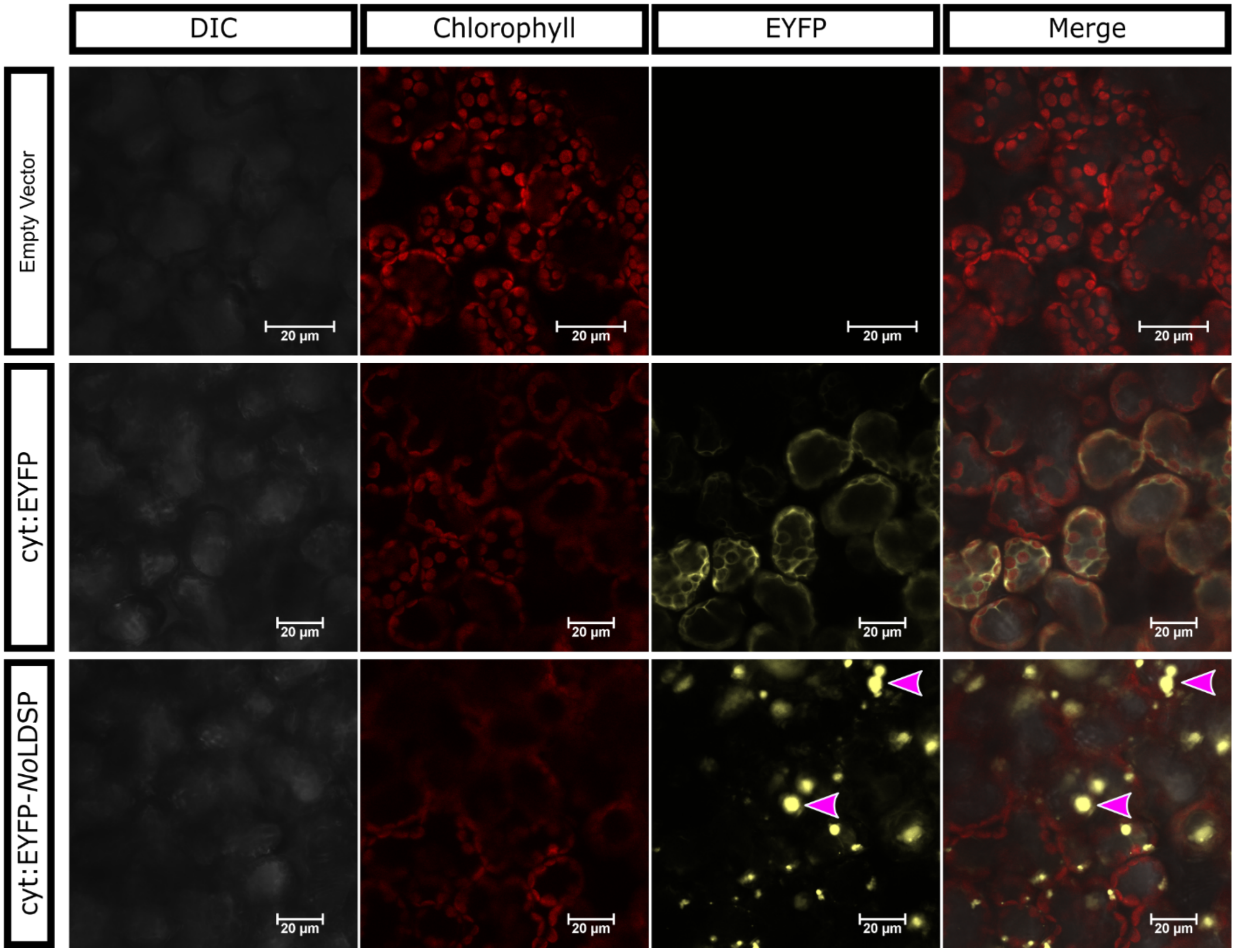

Figure S2. Confocal microscopy comparing cytosolic EYFP and EYFP-*No*LDSP. EYFP fluorescence and chlorophyll autofluorescence were measured with excitation:emission wavelengths of 513.9 nm:585 nm and 561 nm:700 nm, respectively. Pink arrows point to EYFP seen aggregating at lipid droplets in the cytosol.

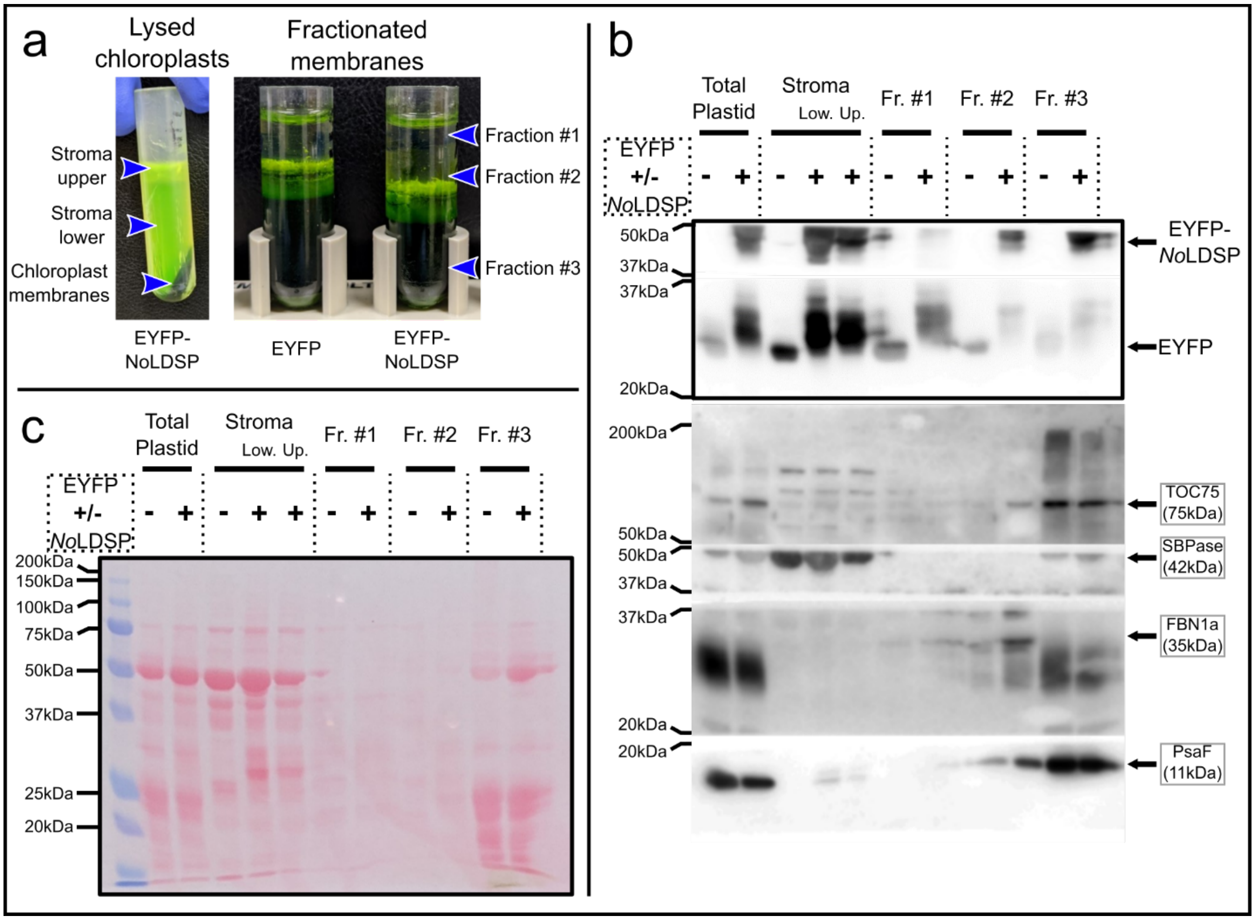

Figure S3. Additional plastid fractionation and western blots demonstrating EYFP-*No*LDSP localization in chloroplast membranes. During chloroplast isolation and fractionation (a), a possible upper layer in the stroma was included for analysis. The nitrocellulose membrane was cut after Ponceau S dye staining (c) to form fragments which could be visualized with each fraction marker by the indicated antibodies (b). The 20 – 37 kDa and 37 kDa – 50 kDa fragments in (b) were first visualized by the fraction specific antibody then washed and re-visualized with anti-GFP. Each lane is indicated as uninfiltrated, wild type plants (WT) or the presence of EYFP with (+) or without (-) fusion to *No*LDSP.

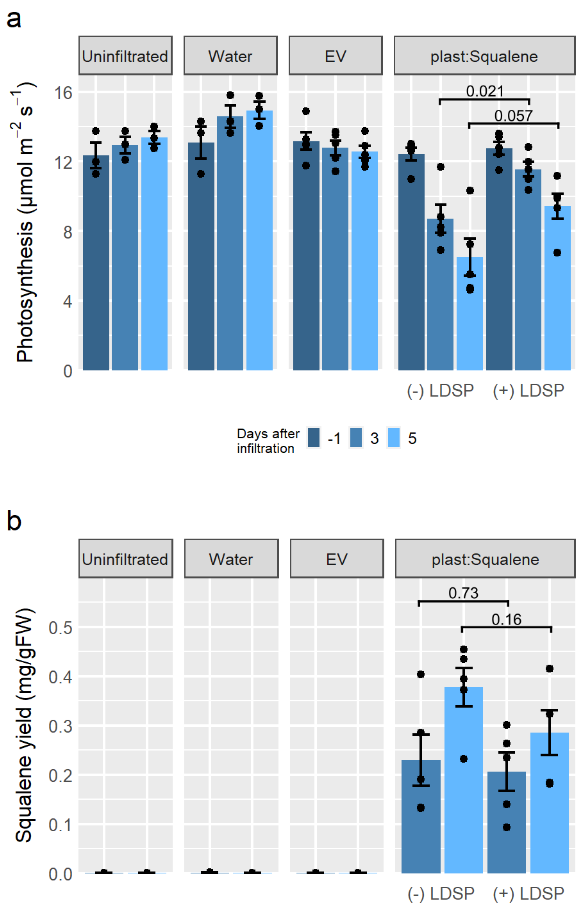

Figure S4. Comparison of the effects of transient expression of plastid targeted squalene pathways in leaves and subsequent effects on photosynthesis compared to controls. Alongside plants expressing the plast:Squalene pathway with (+LDSP) and without (-LDSP) *No*LDSP scaffolding, controls were included for uninfiltrated plants, plants infiltrated with water + 200 µM acetosyringone, and plants infiltrated with *Agrobacterium* harboring the pEAQ-HT empty vector (EV). Black circles show individual data points and bars represent means ± standard error. n = 4 plants per treatment. Individual *t* test statistical comparisons between means are shown by brackets and the indicated p-value.

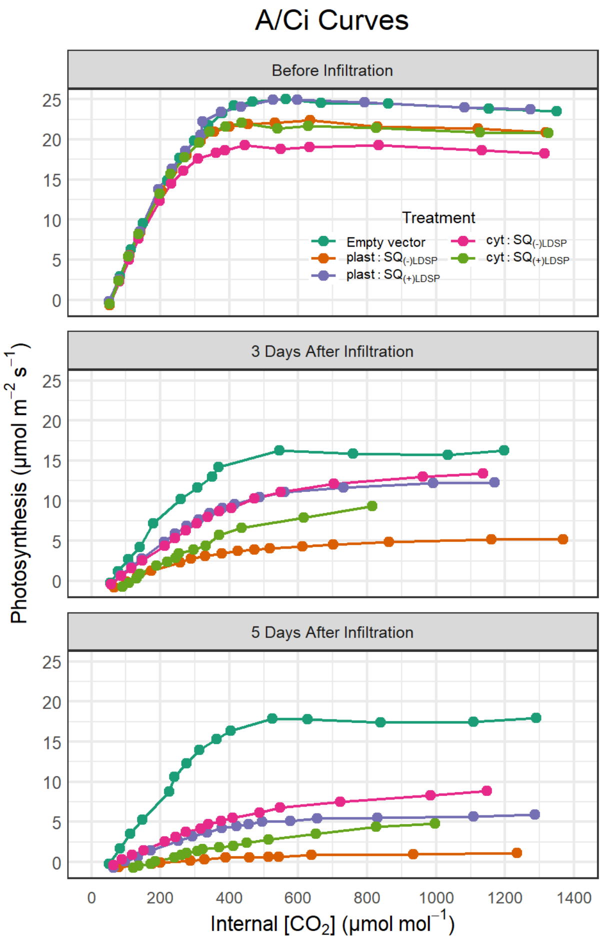

Figure S5. *A*/*C*$_i$ curves comparing plastid targeted and cytosolic squalene pathways, with and without *No*LDSP scaffolding. Each curve was generated from a representative plant for each treatment indicated by color. Data points in each curve represent photosynthesis measured at the indicated internal CO_2 concentration under a saturating light intensity of 1000 µmol m^{-2} s^{-1}. Maximum carboxylation rate (V_{cmax}), maximum rate of electron transport (*J*), and triose phosphate utilization rate (*TPU*) determined by fitting the Farquhar-von Caemmerer-Berry biochemical model of photosynthesis to *A*/*C*$_i$ curves, are presented in Table S2.

References:

(1) Sharkey, T. D.; Bernacchi, C. J.; Farquhar, G. D.; Singsaas, E. L. Fitting Photosynthetic Carbon Dioxide Response Curves for C3 Leaves. *Plant Cell Environ.* **2007**, *30* (9), 1035–1040. https://doi.org/10.1111/j.1365-3040.2007.01710.x.

(2) Sharkey, T. D. What Gas Exchange Data Can Tell Us about Photosynthesis. *Plant Cell Environ.* **2016**, *39* (6), 1161–1163. https://doi.org/10.1111/pce.12641.

(3) Gregory, L. M.; McClain, A. M.; Kramer, D. M.; Pardo, J. D.; Smith, K. E.; Tessmer, O. L.; Walker, B. J.; Ziccardi, L. G.; Sharkey, T. D. The Triose Phosphate Utilization Limitation of Photosynthetic Rate: Out of Global Models but Important for Leaf Models. *Plant Cell Environ.* **2021**, *44* (10), 3223–3226. https://doi.org/10.1111/pce.14153.
